# Unit Cost of Medical Services at Different Hospitals in India

**DOI:** 10.1371/journal.pone.0069728

**Published:** 2013-07-23

**Authors:** Susmita Chatterjee, Carol Levin, Ramanan Laxminarayan

**Affiliations:** 1 Research and Policy, Public Health Foundation of India, New Delhi, India; 2 Department of Global Health, University of Washington, Seattle, Washington, United States of America; 3 Center for Disease Dynamics, Economics and Policy, Washington, D. C., United States of America; 4 Princeton Environmental Institute, Princeton University, Princeton, New Jersey, United States of America; UNAIDS, Switzerland

## Abstract

Institutional care is a growing component of health care costs in low- and middle-income countries, but local health planners in these countries have inadequate knowledge of the costs of different medical services. In India, greater utilisation of hospital services is driven both by rising incomes and by government insurance programmes that cover the cost of inpatient services; however, there is still a paucity of unit cost information from Indian hospitals. In this study, we estimated operating costs and cost per outpatient visit, cost per inpatient stay, cost per emergency room visit, and cost per surgery for five hospitals of different types across India: a 57-bed charitable hospital, a 200-bed private hospital, a 400-bed government district hospital, a 655-bed private teaching hospital, and a 778-bed government tertiary care hospital for the financial year 2010–11. The major cost component varied among human resources, capital costs, and material costs, by hospital type. The outpatient visit cost ranged from Rs. 94 (district hospital) to Rs. 2,213 (private hospital) (USD 1 = INR 52). The inpatient stay cost was Rs. 345 in the private teaching hospital, Rs. 394 in the district hospital, Rs. 614 in the tertiary care hospital, Rs. 1,959 in the charitable hospital, and Rs. 6,996 in the private hospital. Our study results can help hospital administrators understand their cost structures and run their facilities more efficiently, and we identify areas where improvements in efficiency might significantly lower unit costs. The study also demonstrates that detailed costing of Indian hospital operations is both feasible and essential, given the significant variation in the country’s hospital types. Because of the size and diversity of the country and variations across hospitals, a large-scale study should be undertaken to refine hospital costing for different types of hospitals so that the results can be used for policy purposes, such as revising payment rates under government-sponsored insurance schemes.

## Introduction

In health care systems, hospitals provide primary care, serve as referral institutes for higher-level care, and train health care workers. Those benefits are costly [1], however: hospitals require more human and financial resources than any other institution in the sector [2]. A major World Bank study found that the share of public sector health resources consumed by hospitals in developing countries ranged from 50 to 80 percent [3]. In both developed and developing countries, hospitals are viewed as vital and necessary community resources that should be managed for the benefit of the community. Hospital managers, who must provide health care services that the community needs at an acceptable level of quality and at the least possible cost [4], therefore need information on the actual cost of the services they provide.

Information about hospital costs is also needed to inform many types of policy decisions. For example, cost information can help health planners allocate resources to facilities and services [5], introduce or set user fees [6], assess the comparative efficiency of health care services across settings [7], and determine budgets to run health services. In developed countries, hospital costs are important for establishing repayment rates; in low- and middle-income countries, cost information is important for determining reimbursement by social security systems. Nevertheless, very few detailed studies have been carried out on the economics of hospitals in low- and middle-income countries [8], [9]. Recognising the need to make unit cost information available on a country-specific and hospital-level basis, WHO collated data on unit costs from countries and hospitals as part of its WHO-CHOICE project [8]. The study revealed a dearth of unit cost data for health care services, especially in low- and middle-income countries.

In India, government health care services are organised into several levels. Primary health care is provided through a network of 146,036 health subcentres, 23,458 primary health centres (PHCs), and 4,276 community health centres (CHCs) [10]. The subcentres are the most peripheral health units and the first contact point between the health care system and the community. They provide basic drugs for minor ailments and meet people’s essential health needs. PHCs are the first contact between the village community and a medical officer. They act as referral units for six subcentres and have 4 to 6 beds for patients. The CHCs have 30 inpatient beds with one operating theatre, plus x-ray, labour room, and laboratory facilities. At the district level, the government maintains a 150-bed civil or district hospital in the main town and a few other hospitals and dispensaries spread over other towns and larger villages. Tertiary care is specialised consultative health care, usually provided for inpatients following referral from primary or secondary health professionals, in an institution that has personnel and facilities for advanced labwork and imaging as well as highly skilled clinical management [11]. Apart from the government facilities, a significant part of health care is provided by the private sector. Currently, the private sector delivers about 80 percent of all outpatient care and about 60 percent of all inpatient care [10].

Cost studies exist in India for different levels of health care services and providers but are either specific to a particular disease (e.g., typhoid) or based on a specialty (e.g., paediatric care) or service provider (e.g., primary health centre). For example, Anand et al. (1993) estimated the cost incurred by a primary health care centre in northern India during 1991–92 [12]. Krishnan et al. (2005) tried to estimate the cost of outpatient and inpatient paediatric health services provided by the All India Institute of Medical Sciences at all three levels–primary, secondary, and tertiary–for 1999 [13]. Treatment cost of typhoid fever at two hospitals in Kolkata was estimated by Sur et al. (2009) [14]. Mathur et al. (2010) determined the unit cost of curative care provided at primary health care centres in Ahmedabad [15].

To the best of our knowledge, no comprehensive study has been carried out in Indian hospitals to calculate the operating costs of the hospitals as well as the unit costs of the medical services they provide. The present study sought to estimate unit costs of the most basic services provided at different levels or types of hospitals in India. Hence, for all five study hospitals, we calculated (a) cost per outpatient department (OPD) visit; (b) cost per inpatient (IPD) stay (i.e., cost per bed-day); (c) cost per emergency room visit; and (d) cost per surgery.

## Materials and Methods

### Study Design

We estimated the total operating costs of the study hospitals and the unit costs of medical services from a provider perspective. *Unit cost* refers to the cost of providing a single good or service. It can be calculated at various levels of health care provision–at the intermediate service level (e.g., cost per laboratory test) or at the final cost centre level (i.e., cost per inpatient stay or outpatient visit). In addition to calculating the unit cost of OPD visit, IPD stay, emergency room visit, and surgery, we also calculated the costs of some other medical services for individual hospitals, such as cost per laboratory test, cost per admission or bed-day at the intensive care unit, cost per surgery at the orthopaedic, ophthalmic, gynaecology operating theatres, and cost per visit at the physiotherapy and other units.

### Study Hospitals

Five hospitals of different types were chosen for this study based on their willingness to cooperate and the accessibility of hospital data: a 57-bed charitable hospital, a 200-bed private hospital, a 400-bed district hospital, a 655-bed private teaching hospital, and a 778-bed tertiary care teaching hospital. The district and tertiary care teaching hospitals are government hospitals; the charitable hospital is funded by a charitable trust. These hospitals were from four states in India – two in the north and three in the south. The private, charitable, and tertiary care hospitals serve urban populations, the district hospital serves a semi urban area, and the private teaching hospital serves a rural population.

### Data Collection

Annual data were collected for April 2010 through March 2011 (financial year 2010–11) to account for seasonal variations, since each hospital is affected by these factors differently. The main sources of data were the hospitals’ activity and accounting reports. Comprehensive information about human resources in each hospital was taken from the hospital payroll and confirmed by the hospital administrators. Data on such variables as outpatient visits, admissions, and bed-days were collected from the medical records section. Operating theatre statistics were taken from the theatre register. Other activity statistics, like number of laboratory tests and emergency room visits, were taken from the individual departments’ registers. Annual recurrent expenditures, which included salaries, drugs and medical supplies, laboratory, radiology materials, fuel and lubricants, office supplies, maintenance and cleaning, communications, water, electricity, telephone, and Internet, were collected from the annual expenditure report of the hospital.

### Costing Method

The unit costs of medical services have been calculated using the standard costing method [16]. The first step is organisation analysis and cost centre classification. In this step, the study hospitals were divided into several patient care cost centres (PCCs) and supportive cost centres (SCCs). PCCs, such as the inpatient department, outpatient department, and operating theatre, are responsible for direct patient services. SCCs provide support for patient care through administration, laundry, kitchen, transport, and other units.

Next, the direct cost of each cost centre was calculated by summing human resources, capital, and materials costs. Human resources costs include salaries and fringe benefits. For staff who work in more than one cost centre, human resources costs were apportioned based on the working time in each cost centre, as reported by the cost centre supervisors [4]. For the teaching hospitals, we considered only the proportion of staff time spent on patient care–that is, we excluded the proportion of time spent on teaching. For staff categories like nurses and ground-level support staff (group D staff), we used duty rosters to allocate their working time and associated salaries and fringe benefits. Capital costs include annualised discounted depreciation of building, vehicles, equipment, and furniture and the opportunity cost of land. Using the straight-line depreciation approach, we assumed that the services from the capital items were divided equally over the useful life of the capital items. The depreciation cost was calculated using the economic approach, also known as equivalent annual depreciation cost, defined as “an average combination of depreciation cost and interest on undepreciated portion over the useful life of the capital item” [17]. The interest on the undepreciated portion of the capital item was calculated based on the concept of the opportunity cost of money spent in advance for that portion. The interest was calculated for the whole period of the useful life of the capital item, then discounted to the time of analysis. An annuity factor was developed for this purpose. In the calculation, we used replacement cost instead of original cost. Replacement cost adjusts the original cost with an inflation adjustment factor, which is calculated using the consumer price index factor [18].

Some hospitals list fixed assets with the original purchase price and year of purchase. These lists were used where available; otherwise, the fixed rates in recent government contracts for purchasing equipment, instruments, and furniture were used. Examples of items on the equipment list include operating table, operating theatre lighting, Boyle’s trolley, cautry machine, patient examination table, x-ray, ultrasound, ECG machine, C arm, defibrillator, ventilator, cardiac monitor, nebulizer, suction apparatus, radiant warmer, pulse oxymeter, diathermy, autoclave, centrifuge, microscope, and water bath. The instruments list includes forceps, scissors, needle holders, and retractors. The furniture list includes beds, almirah (wardrobes), tables, chairs, lockers, and patient trolleys. The useful life of buildings and structure was considered 20 years; the useful life of other capital items was assumed to be 5 years [19]. A 3 percent discount rate was used to calculate the cost of depreciable assets and the opportunity cost of land [20]. Materials costs cover drugs, medical supplies, office supplies, laboratory, and radiology materials as well as utilities (water, telephone, electricity, Internet).

After deriving the direct cost of each cost centre, we allocated the direct cost of SCCs to PCCs via the simultaneous equation method. We made full adjustment for the interaction of overhead departments, and we solved a set of simultaneous linear equations to determine the allocations [16]. After being allocated to PCCs, the direct costs of SCCs are known as the indirect costs of PCCs. These indirect costs include all costs that could not be allocated directly to final cost centres. In this way, the unit cost includes not just direct costs but also the overhead costs incurred in admitting and treating a patient. In all our study hospitals, the common overhead departments are the administration, nursing administration, laundry, kitchen, maintenance, transport, blood bank, and store. However, because each PCC’s use of indirect costs is unknown, we needed to devise some rule or criterion to allocate these costs. Several studies use floor area to distribute water, electricity, and cleaning services, and bed-days to distribute meal charges. We chose the allocation criteria that would be appropriate for our study hospitals based on either the literature or our knowledge about the particular hospital. The allocation criteria used in this study are presented in Table 1.

**Table 1:** Basis for allocation of indirect costs to patient care cost centres.

*Item*	*Allocation criterion*	*Source*
Administration	Full-time equivalent ofall personnel	[30], [31], [32]
Nursing administration	Nursing full-time equivalent	Present study
Telephone	Full-time equivalent ofall personnel	[33], [34]
Laundry	Estimated actual use	[35]
Office expenses	Full-time equivalent ofall personnel	Present study
Electricity	Floor area	[27], [32]
Water	Floor area	[32]
Cleaning, sanitation	Floor area	[36]
Meal charges	Bed-days	[32], [35], [36]
Maintenance	Estimated actual cost	Present study
Transport	Estimated actual use	[30], [31]
Central sterilization	Items sterilized	Present study
Central store	Total cost of drugs andmaterials	Present study
Medical records section	Admissions	[32], [37], [38]

The direct and indirect costs of each PCC were then added to get the full cost of each PCC. To calculate the unit cost of medical services, we used the average cost method. For example, to find the unit cost per outpatient visit, the full cost of the outpatient department was divided by the total number of outpatient visits at the hospital during the whole year.

## Results

The output of the five hospitals during the study period is presented in Table 2. From the number of visits in the outpatient department, we estimated the average case load per day for the study hospitals. The tertiary care hospital had the highest case load (average 1,045 visits per day), and the charitable hospital, the lowest (average 84 visits per day). One of the efficiency indicators of hospitals is the bed occupancy rate: hospitals with 80 percent occupancy rate are assumed to be efficient. We found that except for the private teaching hospitals, none of the study hospitals achieved an 80 percent occupancy rate during our study period, and the rate was only 42 percent in the charitable hospital. The number of surgeries performed in the study hospitals is reported for general operating theatres only. Apart from general operating theatres, the study hospitals have special operating theatres as well; for example, the tertiary care hospital has orthopaedics, ophthalmology, and cardiothoracic operating theatres, the private hospital has a cardiothoracic operating theatre, and the private teaching hospital has a gynaecology operating theatre; however, the outputs of these operating theatres are omitted from Table 2.

**Table 2:** Output of study hospitals, April 2010–March 2011.

*Output*	*Charitable hospital*	*Private hospital*	*District hospital*	*Private teaching hospital*	*Tertiary care hospital*
Beds	57	200	400	655	778
Outpatient visits	29,829	51,169	293,119	281,756	369,768
Total admissions	2,552	5,925	25,871	19,139	205,949
Occupancy rate	42.12%	59.41%	65.06%	80.34%	72.44%
Total emergency visits	3,696	1,276	12,670	13,642	134,594
Surgeries performed in generaloperating theatre	319	2,508	3,623	2,768	3,219

Table 3 presents the staffing of the study hospitals for the financial year 2010–11. Apart from salaried staff, some hospitals also hired contract staff during the study period. For example, the district hospital had 24 doctors (including 3 in contract service), 98 nurses (including 22 in contract service), 36 paramedical staff (including 3 in contract service), and 90 nonclinical support staff (including 22 in contract service). The private hospital also hired nursing and ground-level support staff on a contract basis during this period. According to the 1993 World Development Report, for quality care, the nurse-to-doctor ratio should be at least 2∶1; a ratio of 4∶1 or better is considered satisfactory [21]. However, except for the district hospital, none of the study hospitals maintained the minimum ratio of 2∶1.

**Table 3:** Staffing in study hospitals, April 2010–March 2011.

	*Total* *staff*	*Doctors*	*Nurses*	*Support* *staff*
Charitable hospital	108	12	22	74
District hospital	248	24	98	126
Tertiary care hospital	1,067	237	212	618
Private hospital	671	103	135	433
Private teaching hospital	620	139	107	374

**Figure 1:**
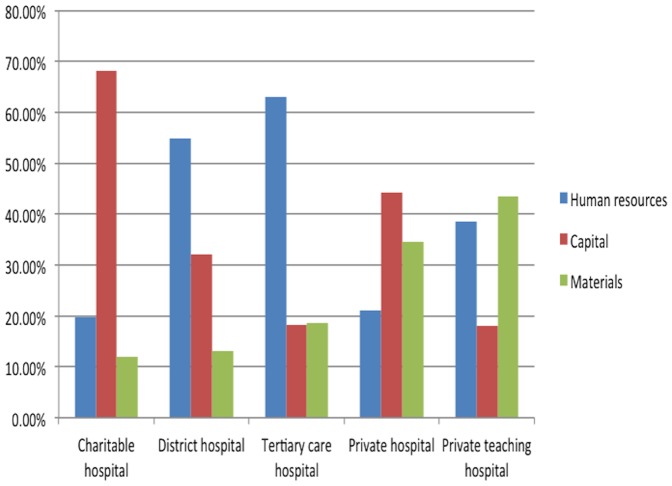
Contribution of different cost components in total costs of study hospitals, April 2010–March 2011. The major cost component for the district and tertiary care hospitals is human resources; for the charitable and private hospitals, it is the capital cost; for the private teaching hospital, it is materials cost.

**Figure 2:**
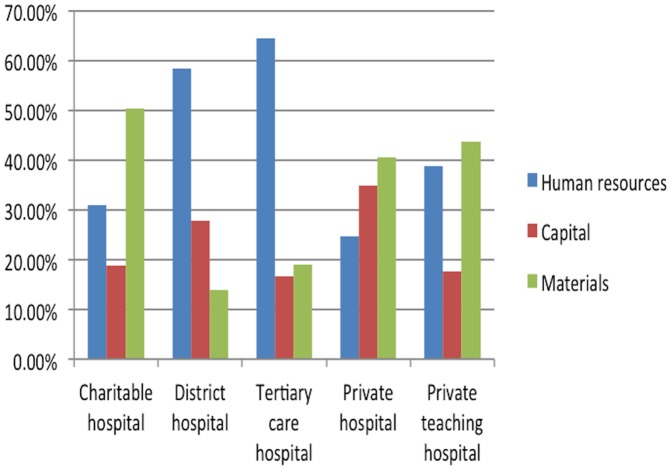
Costs of study hospitals without land cost, April 2010–March 2011. When land cost (which is beyond control of the hospital administrators) is excluded, human resources cost is the main component of total operating cost for the government hospitals, and materials cost becomes the main component for all other hospitals.

We calculated the percentages of human resources, capital, and materials costs in the total operating cost of each hospital. The results are presented in Figure 1. The major cost component for the district and tertiary care hospitals was human resources, for the charitable and private hospitals it was the capital cost, but for the private teaching hospital it was materials cost. If we break down capital cost into equipment, instruments, furniture, building, and land, we obtain very different percentages for the five hospitals (Table 4): land cost was the largest component for the charitable hospital, equipment and building costs shared almost same percentages in the district hospital, equipment cost was the biggest item for both teaching hospitals, and building cost was the highest component for the private hospital. Because land cost is beyond control of the hospital administrators, we did a recalculation excluding land cost. The results are presented in Figure 2. We found that as before, human resources cost was the main component of total operating cost for the government hospitals, but the materials cost became the main component for all other hospitals when land cost was excluded.

**Table 4:** Capital costs of study hospitals, April 2010–March 2011.

	*Total capital cost (INR)*	*Equipment, furniture, instruments*	*Building*	*Land*
Charitable hospital	29,687,483	9.57%	37.76%	52.67%
District hospital	38,584,039	40.08%	41.15%	18.77%
Tertiary care hospital	96,251,015	74.49%	14.19%	11.32%
Private hospital	316,288,355	15.04%	52.35%	32.61%
Private teaching hospital	43,017,966	92.76%	4.86%	2.37%

USD 1 = INR 52.

Materials cost ranged from 12 to 43 percent of the total operating costs of the hospitals. If we break down materials cost into drugs and medical supplies, utilities (electricity, water, telephone, Internet), maintenance, and “other” (laboratory, radiology materials, printing and stationery, laundry, kitchen), we again obtain very different percentages for the study hospitals (Table 5). The drugs and materials supplies category was the biggest item for the tertiary care and private hospitals, but utilities had almost the same share as drugs and medical supplies for the charitable hospital. For the district and the private teaching hospitals, the “other” category accounted for the highest share of materials cost.

**Table 5:** Materials costs of study hospitals, April 2010–March 2011.

	*Total material cost* *(INR)*	*Drugs and medical supplies*	*Utilities*	*Maintenance*	*Other*
Charitable hospital	5,253,171	39.32%	40.70%	5.23%	14.75%
District hospital	15,788,857	19.54%	17.83%	15.38%	47.25%
Tertiary care hospital	98,484,661	60.49%	8.90%	5.21%	25.40%
Private hospital	247,934,158	74.64%	6.02%	10.18%	9.16%
Private teaching hospital	103,974,275	25.78%	10.20%	17.53%	46.49%

Utilities include electricity, water, telephone and Internet.

“Other” includes laboratory, radiology materials, printing and stationery, laundry, kitchen, and all other expenses.

USD 1 = INR 52.

Below we present the operating costs of different PCCs and unit costs for different medical services. Table 6 displays the unit cost of basic services for all study hospitals, and Table 7 shows unit cost of several other (specialised) medical services.

**Table 6:** Unit cost of basic medical services, April 2010–March 2011.

*Cost centre*	*Total cost (INR)*	*Output*	*Unit*	*Unit cost (INR)*
**Charitable hospital**				
OPD	3,427,796	29,829	Visits	115
IPD	17,166,648	8,764	Bed-days	1,959
Emergency	1,421,2450	3,696	Visits	385
Major OT	8,750,188	319	Cases	27,430
**Total operating cost, INR 43,543,262**				
**District hospital**				
OPD	27,488,691	293,119	Visits	94
IPD	32,559,127	82,602	Bed-days	394
Emergency	12,657,103	12,670	Visits	999
Major OT	9,436,319	3,623	Cases	2,605
**Total operating cost, INR 120,388,582**				
**Tertiary care hospital**				
OPD	89,522,217	369,768	Visits	242
IPD	126,424,853	205,949	Bed-days	614
Emergency	67,164,970	134,594	Visits	499
Major OT	26,918,158	3,219	Cases	8,362
**Total operating cost, INR 527,923,769**				
**Private hospital**				
OPD	113,231,544	51,169	Visits	2,213
IPD	188,115,015	26,888	Bed-days	6,996
Emergency	27,910,526	1,276	Visits	21,873
Major OT	60,232,583	2,508	Cases	24,016
**Total operating cost, 715,449,780**				
**Private teaching hospital**				
OPD	53,032,830	281,756	Visits	188
IPD	58,660,813	170,080	Bed-days	345
Emergency	10,742,927	13,642	Visits	787
Major OT	47,598,213	2,768	Cases	17,196
**Total operating cost, INR 239,552,971**				

IPD = inpatient department.

OPD = outpatient department.

OT = operating theatre.

USD 1 = INR 52.

**Table 7:** Unit cost of other medical services, April 2010–March 2011.

*Cost centre*	*Total cost (INR)*	*Output*	*Unit*	*Unit cost (INR)*
**Charitable hospital**				
Laboratory	3,217,667	NA	Cases	–
Radiology	1,637,837	1,990	Cases	823
Pharmacy	947,510	9,539	Prescriptions	99
NICU	5,087,475	778	Admissions	6,539
**District hospital**				
Emergency OT	1,021,764	7,718	Cases	132
Eye OT	2,930,282	1,239	Cases	2,365
IPP OT	1,304,618	386	Cases	3,380
Laboratory	8,825,395	47,326	Tests	186
Pharmacy	3,670,730	293,251	Prescriptions	13
Physiotherapy	904,952	5,662	Visits	160
ICU	3,451,709	440	Admissions	7,845
NICU	8,788,146	1,118	Admissions	7,861
Dialysis unit	3,958,777	1,781	Admissions	2,223
Labour ward	3,390,972	2,776	Cases	1,222
**Tertiary care hospital**				
OPD: medicine, cardiology	33,096,830	129,769	Visits	255
OPD: surgery	19,499,977	84,059	Visits	232
OPD: eye	15,782,112	53,362	Visits	296
OPD: orthopaedics	18,354,932	90,790	Visits	202
IPD: medicine	39,731,843	61,882	Bed-days	642
IPD: surgery	39,404,129	74,216	Bed-days	531
IPD: eye	8,366,546	7,585	Bed-days	1,103
IPD: orthopaedics	22,980,449	45,808	Bed-days	502
Orthopaedics OT	20,403,797	1,785	Cases	11,431
Emergency OT	15,648,684	4,446	Cases	3,520
*All laboratory*	*48,509,048*	*784,972*	*Tests*	*62*
Microbiology laboratory	12,092,740	87,146	Tests	139
Biochemistry laboratory	13,640,794	613,084	Tests	22
Pathology laboratory	22,775,514	84,742	Tests	269
Radiology	47,410,516	151,015	Tests	314
Physiotherapy	7,683,767	19,260	Visits	399
**Private hospital**				
CTOT	52,506,934	533	Cases	98,512
Laboratory	36,868,238	NA	Tests	–
Radiology	31,054,870	NA	Tests	–
Pharmacy	65,879,948	NA	Prescriptions	–
SICU	19,091,664	1,623	Bed-days	11,763
CTICU	29,200,747	1,313	Bed-days	22,240
RICU	25,052,733	1,445	Bed-days	17,338
MICU	27,541,425	1,768	Bed-days	15,578
ICCU	24,794,259	2,992	Bed-days	8,287
Dialysis unit	13,969,294	3,385	Visits	4,127
**Private teaching hospital**				
Gyn OT	12870851	2,055	Cases	6,263
Laboratory	9,835,855	388,102	Tests	25
Radiology	20,872,248	61,646	Tests	339
Pharmacy	3,004,746	136,214	Prescriptions	22
Labour room	4,579,199	1,918	Cases	2,387
MICU	7,800,575	3,724	Bed-days	2,095
NICU	4,393,140	5,333	Bed-days	824
SICU	6,161,573	2,340	Bed-days	2,633

CTICU = cardiothoracic intensive care unit.

CTOT = cardiothoracic operating theatre.

Gyn OT = gynaecology operating theatre.

ICCU = intensive cardiac care unit.

ICU = intensive care unit.

IPD = inpatient department.

IPP = Indian population project.

MICU = medicine intensive care unit.

NICU = neonatal intensive care unit.

OPD = outpatient department.

OT = operating theatre.

RICU = respiratory intensive care unit.

SICU = surgical intensive care unit.

USD 1 = INR 52.

The OPD visit cost ranged from Rs. 94 in the district hospital to Rs. 2,213 in the private hospital (USD 1 = INR 52) (Table 6). Inpatient stay costs were Rs. 345 in the private teaching hospital, Rs. 394 in the district hospital, Rs. 614 in the tertiary care hospital, Rs. 1,959 in the charitable hospital, and Rs. 6,997 in the private hospital. The emergency visit cost was lowest in the charitable hospital (Rs. 385) and highest in the private hospital (Rs. 21,873). Although the average cost of general surgical procedures was Rs. 2,605 in the district hospital, it was Rs. 8,362 in the general surgical operating theatre in the tertiary care hospital. Surgical procedures were very expensive in the private and charitable hospitals–Rs. 24,016 and Rs. 27,430 per procedure, respectively. In the private teaching hospital, the average cost of general surgical procedures was Rs. 17,196.

Table 7 provides unit costs of different specialised services. Of the different wards in the tertiary care hospital, inpatient stay cost was highest in the ophthalmology ward. The OPD visit cost did not differ much across specialties in the tertiary care hospital. Overall, laboratory tests were cheaper in the teaching hospitals (Rs. 25 in the private teaching hospital and Rs. 62 in the tertiary care hospital) than in the district hospital (Rs. 186), whereas physiotherapy was cheaper in the district hospital (Rs. 160) than in the tertiary care hospital (Rs. 399). Average cost per radiology test was almost the same in the two teaching hospitals (Rs. 314 in the tertiary care hospital and Rs. 339 in the private teaching hospital). Cost per admission at the neonatal intensive care unit of the charitable hospital was Rs. 6,539, compared with Rs. 7,861 at the district hospital. Average cost of normal delivery was Rs. 1,222 in the district hospital and Rs. 2,387 in the private teaching hospital.

We also tried to find out the contributions of the OPD, IPD, emergency room, and operating theatre to each hospital’s total operating cost (Table 8). These four cost centres consumed more than 50 percent of the total operating costs of the hospitals during our study period. IPD consumed the largest proportion of resources in all hospitals: 19.99 percent in the private teaching hospital, 20.93 percent in the tertiary care hospital, 26.29 percent in the private hospital, 26.93 percent in the charitable hospital, and 27.04 percent in the district hospital. OPD was the next highest. Within an individual cost centre, human resources accounted for the highest cost share, followed by materials cost, in both the district and tertiary care hospitals. For example, human resources costs contributed 17 percent of the total costs of IPDs in the charitable and private hospitals, 37 percent in private teaching hospital, and about 55 percent in the district and tertiary care hospitals. As expected, equipment accounted for a large share of the costs for the operating theatres of all hospitals. Materials cost contributed a significant share in all cost centres except the operating theatre in the tertiary care hospital, the IPD and operating theatre in the private hospital, and all cost centres in the private teaching hospital.

**Table 8:** Cost of selected medical services as percentage of total cost at study hospitals, April 2010–March 2011.

*Units*	*Human resources*		*Materials*	*Building*	*Land*	*Equipment*			*Indirect cost* *	*Total*
**Charitable hospital**										
OPD		0.72 (3%)	1.12 (5%)	2.16 (11%)	0.96 (5%)	0.16 (1%)			15.24 (75%)	20.37 (100%)
IPD		4.68 (17%)	3.57 (13%)	10.57 (39%)	4.72 (17%)	0.64 (2%)			2.75 (10%)	26.93 (100%)
Emergency		0.68 (21%)	0.18 (5%)	0.54 (16%)	0.24 (7%)	0.06 (2%)			1.57 (48%)	3.26 (100%)
OT		2.21 (11%)	2.80 (14%)	4.16 (21%)	1.86 (9%)	1.80 (9%)			7.26 (36%)	20.10 (100%)
**District hospital**										
OPD		7.10 (31%)	3.49 (15%)	6.57 (29%)	2.99 (13%)	1.31 (6%)			1.38 (6%)	22.83 (100%)
IPD		14.87 (55%)	2.31 (8%)	2.92 (11%)	1.33 (5%)	0.80 (3%)			4.82 (18%)	27.04 (100%)
Emergency		4.31 (41%)	0.40 (4%)	0.31 (3%)	0.14 (1%)	0.04 (0.4%)			5.32 (51%)	10.51 (100%)
OT		3.17 (40%)	0.61 (8%)	0.39 (5%)	0.18 (2%)	2.89 (37%)			0.60 (8%)	7.84 (100%)
**Tertiary care hospital**										
OPD		6.80(41%)	6.37 (39%)	0.13 (0.8%)	0.09 (0.6%)	0.52 (3%)		2.52 (15%)		16.43 (100%)
IPD		11.35 (54%)	5.01 (24%)	0.69 (3%)	0.50 (2%)	0.19 (1%)		3.20 (15%)		20.93 (100%)
Emergency		4.87(38%)	2.91 (23%)	0.38 (3%)	0.27 (2%)	0.96 (7%)		3.34 (26%)		12.72 (100%)
OT		3.20 (63%)	0.30 (6%)	0.04 (0.9%)	0.03 (0.6%)	0.65 (13%)		0.87 (17%)		5.10 (100%)
**Private hospital**										
OPD		4.70 (30%)	1.49 (9%)	3.38 (21%)	2.10 (13%)		1.30 (8%)	2.86 (18%)		15.83 (100%)
IPD		4.43 (17%)	8.04 (30%)	5.41 (21%)	3.37 (13%)		0.31 (1%)	4.74 (18%)		26.29 (100%)
Emergency		1.20 (31%)	0.32 (8%)	0.62 (16%)	0.39 (10%)		0.20 (5%)	1.17 (30%)		3.90 (100%)
OT		2.23 (14%)	7.60 (48%)	1.18 (7%)	0.74 (5%)		1.56 (10%)	2.45 (16%)		15.76 (100%)
**Private teaching hospital**										
OPD		5.57 (31%)	8.45 (47%)	0.23 (1%)	0.11 (0.63%)		3.59 (20%)	0.15 (0.84%)		18.11 (100%)
IPD		7.49 (37%)	10.97 (55%)	0.39 (2%)	0.19 (1%)		0.65 (3%)	0.29 (1%)		19.99 (100%)
Emergency		2.22 (70%)	0.59 (19%)	0.02 (1%)	0.01 (0.32%)		0.27 (8%)	0.08 (2%)		3.18 (100%)
OT		5.93 (34%)	6.95 (40%)	0.01 (1%)	0.01 (0.04%)		4.22 (24%)	0.15 (1%)		17.27 (100%)

* Indirect cost is the direct cost of support after it is allocated to a patient care cost centre.

## Discussion

In what may be the first comprehensive study on the economics of Indian hospitals, we have presented the operating costs and unit costs of medical services at five hospitals of different types. We find that human resources are the largest component of a hospital’s total operating cost for the government’s district and tertiary care hospitals. This is consistent with the findings of the report of the working group on tertiary care institutions in India, which showed that in most states, salaries and wages account for as much as 70 percent of the total health budget [11]. Several international studies on hospital costing also found that human resources constitute the majority of a hospital’s total operating cost [1], [22], [23], [24]. Human resources cost is less at the charitable and the private hospital because the salary structure at these hospitals is lower than the prevailing market rate (except for some doctors). Even though the human resources costs are high in the district and tertiary care hospitals, however, the nurse-to-doctor ratio is below the recommended minimum of 2∶1. In the district hospital this ratio is more than 2∶1, but 22 nurses are on contract service and are appointed specifically for the neonatal intensive care unit and emergency. Their pay is also much lower than that of the regular nursing staff. The shortage of nursing staff in the district hospital is evident from the duty rosters: one nurse is responsible for three inpatient wards at night. None of the other hospitals were able to achieve the minimum target of 2∶1, which raises questions about the quality of care. However, even though the tertiary care hospital and the private teaching hospital had a shortage of nursing staff, nursing students provided support for different activities, and thus the effective quality of care might be better than at the hospitals that had no such voluntary services. We also found that ground-level nontechnical support staff (group D staff) were performing multiple tasks simultaneously. Such understaffing can affect efficient functioning, and hospital administrators should be aware of the problem.

Because a significant proportion of hospital costs is fixed, the intensity with which resources are used is likely to influence unit costs. The first step in elucidating the unit cost is to examine bed occupancy rates [9]. The overall occupancy rate did not exceed 80 percent at any of the hospitals except the private teaching hospital during our study period and was only 42 percent in the charitable hospital. Because occupancy rate affects the inpatient stay cost, we recalculated the inpatient stay cost using an 80 percent occupancy rate, as recommended by WHO [20]. At 80 percent occupancy, the inpatient stay cost at the charitable hospital then falls by almost half, from Rs. 1,959 to Rs. 1,031. Similarly, for the district hospital, the inpatient stay cost falls from Rs. 394 to Rs. 320; for the tertiary care hospital, from Rs. 614 to Rs. 557; and for the private hospital, from Rs. 6,996 to Rs. 5,195. At the tertiary care hospital, the occupancy rates in the medicine, surgery, and orthopaedics wards were 80 percent and above during our study period, but in the ophthalmology ward the rate was only 31 percent, which lowered the hospital’s overall occupancy rate to 72 percent. The inpatient stay cost at the ophthalmology ward (Rs. 1,103) is much higher than in other wards (e.g., Rs. 502 in the orthopaedics ward). The occupancy rate at the ophthalmology ward could be low because many ophthalmologic procedures are day care services; however, the hospital administrator may want to consider more efficient utilisation of a ward whose bed occupancy rate is so low. At an 80 percent occupancy rate, the private hospital’s inpatient stay cost declines but is still much higher than all other hospitals. The emergency visit cost at the private hospital is also exceptionally high compared with other hospitals. Although this hospital has a well-equipped emergency department, only 1,276 visits occurred during the whole study period. The private hospital administrator should examine the reasons for low caseloads in emergency department. Further, the OPD visit in this hospital is also the costliest among all study hospitals. One reason for the overall high cost at the outpatient and inpatient departments of the private hospital could be its focus on specialised cases, for which cases may be few; however, the private hospital administrator should determine whether caseloads or admissions could be increased to improve the unit cost. Similarly, the cost per procedure at the charitable hospital is also very high; this is because only 319 procedures were conducted during the whole study period. Therefore, the charitable hospital administrators should also examine the reasons of lower case loads at the operating room. Our study suggests that the overall unit costs per inpatient stay and outpatient visit give reasonable estimates of the costs for different hospitals and different wards and OPDs within individual hospitals, and these indicators can therefore be used to monitor efficiency.

We have compared our findings with other developing country experiences. A common pattern in developing countries is hospitals’ very substantial outpatient functions. Mills (1990) noted that outpatient care absorbed approximately 20 percent of current hospital expenditure in seven developing countries’ hospitals [25]. Plaetse et al. (2005) also found that 23 percent of the total cost of a district hospital was for the outpatient department [26]. Our study hospitals consumed, on average, 19 percent of all costs for outpatient functions, which is in line with other studies. However, inpatient departments consumed more resources than outpatient services (average 24 vs. 19 percent).

As mentioned earlier, bed occupancy rate explains some variation in unit cost, but it is important to distinguish the costs that can be controlled at the individual hospital level from the costs over which the hospitals have no control. For example, the land and building cost of the charitable and private hospitals constitute a significant proportion of the total hospital cost because the local value of land, and hence the rental value, is very high. Even though this cost is beyond their control, the hospitals’ administrators could aim for better utilisation of the space. At both teaching hospitals, the cost of equipment, furniture, and instruments is a significant portion of the total capital cost. The hospital administrators should therefore look at the price and quantity of equipment, furniture, and instruments used in the hospital. Drugs and medical supplies account for 60 percent of the total materials cost at the tertiary care hospital, and 75 percent at the private hospital. The administrators might therefore seek efficiencies in purchasing and stocking drugs and medical supplies. Drugs and medical supplies account for only about 20 percent of the total materials cost in the district hospital, and about 26 percent in the private teaching hospital. But “other” costs (laboratory, radiology materials, printing and stationery, etc.) contribute a significant proportion, about 47 percent, of total materials cost in the district and the private teaching hospitals. Their administrators may want to consider a more rational use of such materials and look for efficiencies in maintenance.

The present study thus identifies areas where improvements in efficiency might yield significant cost savings. Although the scope for increasing the number of admissions and patient visits is limited, the study hospitals could investigate whether improved services might increase admissions and hence the bed occupancy rate. For example, the district hospital’s general surgery department is managed by only one surgeon. Appointing more general surgeons would improve utilisation of the operating theatre.

The study also demonstrates that detailed costing of hospital operations in India is feasible. The biggest challenge lies in collecting data from the hospitals. Although accounts data can be accessed relatively easily, obtaining accurate activity statistics, stock-related data, and price information is difficult. Mills et al. (1993) encountered similar problems in accessing accurate activity statistics in Malawi [9]. Our study hospitals (especially the government hospitals) do not calculate their bed occupancy rates and hence do not have these statistics readily available. To calculate occupancy, researchers must go through the daily census form and manually record the number of beds occupied every day for the duration of the study period. The operating theatres maintain the statistics for the total number of surgeries performed every month but do not keep records of the types of surgeries performed; the researcher must go through the operating theatre register and count the surgeries by type for the period of interest. Moreover, some hospitals do not have proper stock registers of equipment, furniture, or instruments, or they do not track the distribution of drugs and medical supplies among cost centres. Hence, improvements in hospital recordkeeping could help researchers, whose studies could then help the hospitals become more efficient.

Our hospital costing exercise followed the costing methodology used by several other researchers in different countries [1], [14], [27], [28]. However, some limitations of the present study merit comment. First, because the tertiary care hospital was unable to indicate the distribution of drugs and medical supplies among cost centres, we used number of visits and admissions to distribute this cost. Based on the hospital physicians’ opinions, we assumed that patients in the wards consumed three times more drugs and medical supplies than did the patients seen at the OPD or emergency room. Although expert opinion is an accepted method for resource allocation, the resulting figures are not exact. Hence, unit costs of different departments at the tertiary care hospital might be either under- or overestimated. Second, because we did not have access to price data for some equipment and instruments in the ophthalmic operating theatre of the tertiary care hospital, the equipment cost of this department is an estimate. This might affect the unit cost estimate of the ophthalmic department of this hospital. Third, donated items have not been considered in the cost calculation. Shepard et al. (1998) have argued for the inclusion of donated items in cost analyses, since hospitals or wards with more donated items may appear more efficient than their peers, even though their actual efficiency may be the same. Such items can account for a substantial share of hospital resources [4]. Because the study hospitals did not keep any record of donated items, we excluded them from our calculation, but the unit cost estimates of the study hospitals would have been different had they been included. Fourth, quality of services could clearly explain some of the variations in costs but was beyond the scope of this study. Fifth, using duty rosters to allocate nursing and ground-level staff time provides only estimates of time allocation. However, it was impossible to get exact time allocations for these staff categories. Finally, because one goal of this study was to determine the feasibility of doing cost estimates in the Indian health care sector, we chose five hospitals of different types whose administrators would agree to cooperate and provide data. Given the diversity of hospitals in India, our study hospitals might not be representative.

## Conclusion

The present study provides information on the actual cost of providing clinical services in five hospitals of different types in India. The study results have been shared with all hospital administrators so that they can assess their hospitals’ efficiency and make informed hospital planning and management decisions. The results can also be used for policy purposes, such as setting or revising the payment rate. For example, Rashtriya Swasthya Bima Yojana (RSBY, the Indian government-supported health insurance scheme for the people living below the poverty line), pays providers Rs. 500 per day for inpatient stay [29]. This rate includes bed charges (in the general ward), nursing and boarding charges, surgeons, anaesthetists, medical practitioners, consultants’ fees, blood, oxygen, medicines and drugs, x-rays, and diagnostic tests. Our study found that costs per bed-day vary from Rs. 345 to Rs. 6,996 (excluding x-ray and diagnostic tests); hence, this type of costing study can be used to revise the payment rates. Assuming that the provider payment rate might not include capital cost, we did a recalculation excluding capital cost from the operational cost of the study hospitals. We found that the average decline in unit costs of basic medical services (i.e. cost per OPD visit, IPD stay, emergency room visit and surgery) without capital cost was the highest for the charitable hospital (68%) followed by the private hospital (44%), district hospital (33%), private teaching hospital (15%) and tertiary care hospital (13%).

This is a preliminary study on hospital costing in India. Given the size and diversity of the country and variations across hospitals, a large-scale study should be undertaken to gain a better understanding of hospital costing for different types of hospitals and provide more comprehensive information for policy purposes.

## References

[1] Minh HV, Giang KB, Huong DL, Huong LH, Huong NT, et al (2010) Costing of clinical services in rural district hospitals in northern Vietnam. Int J Health Plan M 25: 63–73. 10.1002/hpm.970 19165764

[2] Newbrander W, Barnum H, Kutzin J (1992) Hospital economics and financing in developing countries. Geneva: World Health Organization.

[3] Barnum H, Kutzin J (1993) Public hospitals in developing countries. Baltimore: Johns Hopkins Press.

[4] Shepard DS, Hodgkin D, Anthony Y (1998) An analysis of hospital costs: a manual for managers. Geneva: World Health Organization, HSD Programme.

[5] Green A, Ali B, Naeem A, Vassall A (2001) Using costing as a district planning and management tool in Balochistan, Pakistan. Health Policy Plann 16: 180–186. 10.1093/heapol/16.2.180 11358919

[6] Conteh L, Walker D (2004) Cost and unit cost calculations using step-down accounting. Health Policy Plann 19: 127–135. 10.1093/heapol/czh015 14982891

[7] Adam T, Evans D, Murray C (2003) Econometric estimation of country-specific hospital costs. Cost Eff Resour Alloc 1: 3. 12773218 10.1186/1478-7547-1-3 PMC156022

[8] Adam T, Evans D (2006) Determinants of variation in the cost of inpatient stays versus outpatient visits in hospitals: a multi country analysis. Soc Sci Med 63: 1700–1710. 16769168 10.1016/j.socscimed.2006.04.023

[9] Mills AJ, Kapalamula J, Chisimbi S (1993) The cost of the district hospital: a case study in Malawi. Bull World Health Organ 71: 329–339. 8324852 PMC2393502

[10] Government of India (2010) Annual report to the people on health. New Delhi: Ministry of Health and Family Welfare.

[11] Government of India (2011) Report of the working group on tertiary care institutions for 12th five-year plan (2012–17). New Delhi: Planning Commission.

[12] Anand K, Kapoor SK, Pandav CS (1993) Cost analysis of a primary health centre in northern India. Natl Med J India 6: 160–163. 8401192

[13] Krishnan A, Arora NK, Pandav CS, Kapoor SK (2005) Cost of curative pediatric services in public sector setting. Indian J Pediatr 72: 657–660. 16131769 10.1007/BF02724072

[14] Sur D, Chatterjee S, Riewpaiboon A, Manna B, Kanungo S, et al (2009) Treatment cost for typhoid fever at two hospitals in Kolkata, India. J Health Popul Nutr 27: 725–732. 20099755 10.3329/jhpn.v27i6.4323 PMC2928117

[15] Mathur N, Kedia G, Trivedi A (2010) A comparative study to analyse the cost of curative care at primary health centre in Ahmedabad. Indian J Community Med 35: 153–158. 20606942 10.4103/0970-0218.62585 PMC2888347

[16] Drummond MF, Sculpher MJ, Torrance GW, O’Brien BJ, Stoddart GL (2005) Methods for the economic evaluation of health care programmes. Oxford: Oxford University Press.

[17] Walker D, Kumaranayake L (2002) Allowing for differential timing in cost analyses: discounting and annualization. Health Policy Plann 17: 112–118. 10.1093/heapol/17.1.112 11861593

[18] Riewpaiboon A (2010) Cost analysis in health care. Bangkok: Mahidol University.

[19] Creese A, Parker D (2000) Cost analysis in primary health care: a training manual for programme managers. Geneva: World Health Organization.

[20] Edejer TT-T, Baltussen R, Adam T, Hutubessy R, Acharya A et al.. (2003) Making choices in health: WHO guide to cost-effectiveness analysis. Geneva: World Health Organization.

[21] World Bank (1993) World Development Report. Washington DC: World Bank.

[22] Oostenbrink JB, Van der Woude TB, Van Agthoven M, Koopmanschap MA, Rutten FF (2003) Unit costs of inpatient hospital days. Pharmacoeconomics 21: 263–271. 12600221 10.2165/00019053-200321040-00004

[23] Kruk ME, Wladis A, Mbembati N, Ndao-Brumblay SK, Hsia RY, et al (2010) Human resource and funding constraints for essential surgery in district hospitals in Africa: a retrospective cross-sectional survey. PLos Med 7: e1000242. 20231869 10.1371/journal.pmed.1000242 PMC2834706

[24] Olukoga A (2007) Unit costs of inpatient days in district hospitals in South Africa. Singapore Med J 48: 143–147. 17304394

[25] Mills A (1990) The economics of hospitals in developing countries. Part II: costs and sources of income. Health Policy Plann 5: 203–218.

[26] Plaetse BV, Hlatiwayo G, Eygen LV, Meessen B, Criel B (2005) Costs and revenue of health care in a rural Zimbabwean district. Health Policy Plann 20: 243–251. 10.1093/heapol/czi028 15965036

[27] Riewpaiboon A, Chatterjee S, Piyauthakit P (2011) Cost analysis for efficient management: diabetes treatment at a public district hospital in Thailand. Int J Pharm Pract 19: 342–349. 21899614 10.1111/j.2042-7174.2011.00131.x

[28] Riewpaiboon A, Youngkong S, Sreshthaputra N, Stewart JF, Samosornsuk S et al. 2008. A cost function analysis of Shigellosis in Thailand. Value Hlth 11: S75–S83. 10.1111/j.1524-4733.2008.00370.x 18387071

[29] Rashtriya Swasthya Bima Yojana website (2012) Available: http://www.rsby.gov.in/.Accessed on 21.01.13.

[30] Maggie HR (1992) Financial study of Thimphu General Hospital: recurrent cost analysis and selected options for privatization and user fees. Bhutan: Department of Health Services, Ministry of Social Services, Royal Government of Bhutan.

[31] Maggie HR (1992) Dzongkhag costing study for Tashigang Dzongkhag. Bhutan: Department of Health Services, Ministry of Social Services, Royal Government of Bhutan.

[32] Ministry of Health, Gambia/World Health Organization (1995) Cost analysis of the health care sector in the Gambia. Geneva: World Health Organization.

[33] Wong H (1989) Cost analysis of Niamey Hospital. Bethesda, MD: Abt Associates.

[34] Wong H (1993) Health financing in Tuvalu. Health Financing and Sustainability Project, Technical Report No. 11. Bethesda, MD: Abt Associates.

[35] Lewis M, La Forgia GM, Sulvetta MV (1996) Measuring public hospital costs: empirical evidence from the Dominican Republic. Soc Sci Med 43: 221–243. 8844926 10.1016/0277-9536(95)00364-9

[36] John Snow Inc (1990) Papua New Guinea: health sector financing study project. Final Report: Volume II. Hospital cost study. Boston, MA: John Snow Inc.

[37] Kutzin J (1989) Jamaican Hospital restoration project. Final report. Chevy Chase, MD: Project HOPE.

[38] Russell S, Gwynne G, Trisolini M (1988) Health care financing in St. Lucia and costs of Victoria Hospital. Stony Brook, NY: State University of New York.

